# Exploring the Diversity and Applications of Lactic Acid Bacteria from Tunisian Traditional Fermented Foods

**DOI:** 10.3390/microorganisms14020383

**Published:** 2026-02-05

**Authors:** Sabrine Alebidi, Hana Mallek, Mariagiovanna Fragasso, Vittorio Capozzi, Ferid Abidi, Ines Essid, Giuseppe Spano, Hiba Selmi

**Affiliations:** 1 Laboratory of Protein Engineering and Bioactive Molecules (LIP-MB), National Institute of Applied Sciences and Technology, University of Carthage, Carthage 1054, Tunisia; sabrine.alebidi@etudiant-fst.utm.tn (S.A.); ferid.abidi@insat.ucar.tn (F.A.); hiba.selmi@unifg.it (H.S.); 2 Enhancing Tunisian Natural Heritage and Agriculture Processed Products Through Innovation, UR-PATIO (UR17AGR01), National Institute of Agronomy of Tunisia (INAT), University of Carthage, 43 Avenue Charles Nicole, Tunis 1082, Tunisia; mallek.hana@inat.ucar.tn (H.M.); ines.essid@inat.ucar.tn (I.E.); 3 Department of Agriculture, Food, Natural Science, and Engineering (DAFNE), University of Foggia, 71122 Foggia, Italy; giuseppe.spano@unifg.it; 4 Institute of Sciences of Food Production, National Research Council (CNR) of Italy, c/o CS-DAT, Via Michele Protano, 71121 Foggia, Italy

**Keywords:** LAB, Tunisia, traditional fermented foods, sources, applications, biodiversity, innovative food systems

## Abstract

Tunisian traditional fermented foods represent a valuable cultural heritage transmitted across generations and are highly appreciated by consumers for their distinctive flavours, textures, and nutraceutical value. This review provides the first comprehensive and exclusive overview of lactic acid bacteria (LAB) associated with Tunisian traditional fermented foods, both plant- and animal-based. The overview integrates data across dairy, meat, fish, vegetable, and cereal matrices, highlighting the central role that LAB play in the processing of these foods, driving fermentation and shaping the quality and safety of final products. During fermentation, LAB produce a variety of bioactive metabolites, including organic acids, antimicrobial compounds, exopolysaccharides, enzymes, and vitamins, which enhance food safety, shelf life, nutritional quality, and health-promoting potential. The studies include evidence of LAB’s long history of safe use by humans, including the characterisation of autochthonous strains with protechnological, bioprotective, and probiotic properties, providing candidates for the design of starter, protective and probiotic cultures. By consolidating evidence on the relevance of microbial diversity, this review positions Tunisian LAB as valuable resources for both traditional food valorisation and innovative food system development. Importantly, key knowledge gaps are identified, including the limited application of omics-based tools, insufficient genomic safety assessments, and the lack of systematic analysis linking LAB diversity with the desired attributes to promote innovations. Overall, this review provides a structured framework for the valorisation of Tunisian agrofood heritage, bridging artisanal knowledge with modern food microbiology and offering strategic directions for future research, industrial translation, and sustainable innovation in fermented foods.

## 1. Introduction

Traditional fermented foods and beverages occupy an important place in humans’ diet. It has been reported that fermented foods account for 5% to 40% of the daily diet [1]. In fact, fermented foods provide nutrients and confer beneficial effects, such as managing lactose intolerance, lowering cholesterol, improving immune function, and inhibiting the adherence of some pathogens [2]. In food fermentation, two principles can be applied: the food can be transformed naturally through “spontaneous ferments,” in which the microorganisms are naturally present in the raw food or processing environment, or through the addition of starter cultures, known as “culture-dependent ferments” [3].

In Africa, diets rely principally on fermented foods and other conservation methods [2]. North African countries have a rich culinary tradition, and numerous traditional fermented foods of animal and/or plant origin have been passed down from one generation to the next [4]. Particularly Morocco, Algeria, and Tunisia, which share a rich and diverse array of dietary habits and food cultures. This commonality is manifested in the use of similar ingredients, traditional cooking methods, and the significance of communal meals, all of which are influenced by their shared geographical and historical connections [5]. The vast diversity of traditional artisanal foods results from the blending of dietary traditions brought by ancient civilisations such as the Greeks, Phoenicians, Romans, and Andalusians [6].

Focusing on Tunisia, a North African and Arabic country, it is part of the Mediterranean region. This country is characterised by a rich and unexploited heritage with a wide range of traditional fermented products, including (i) fermented dairy products like leben, raïb, and semen (fermented milk), (ii) fermented plant- and vegetable-based products such as fermented olives, and (iii) fermented meat like kadid [7,8,9,10]. Understanding of the microbial characteristics and fermentation processes of these foods remains limited. Indeed, most fermentation processes were traditionally carried out on a small scale, relying on spontaneous fermentation driven by indigenous microorganisms [11]. These microbial communities not only ensure the safety and stability of foodstuffs but also contribute to their nutritional and functional properties. Among the first Tunisian investigations in this field was one conducted more than 25 years ago; in 1996, Khosrof and colleagues studied the microbiological characteristics of Tunisian milk products (cheeses, yoghurt, and milks) that were fermented spontaneously. As a result, a new *Streptococcus* strain with bacteriocin production ability was isolated and further characterised from a single raw material, e.g., milk. A variety of fermented products can be produced through spontaneous fermentation, relying on naturally present microbes, containers, and environmental conditions (temperature, duration, and humidity) [11]. These abiotic conditions indirectly select the strains of interest for the transformation process, thereby leading to unique taste, texture, and consistency in the final product. Generally, traditional fermented foods contain complex microbial communities that can confer health benefits associated with food consumption [4], such as modulation of gut microbiota, enhancement of immune function, production of antimicrobials and antioxidants, and/or induction of cholesterol-lowering activities [2,12]. Recent studies also indicate that certain probiotic microorganisms and their metabolites may exert anticancer and antitumor effects, further contributing to human health and reinforcing the functional value of fermented foods [13,14,15]. The predominant probiotic bacteria in fermented food are lactic acid bacteria (LAB) from the genera *Lactococcus*, *Pediococcus*, *Lactobacillus*, *Lactiplantibacillus*, *Streptococcus*, and *Leuconostoc* [12]. LAB constitute a diverse group of microorganisms of considerable importance in the food industry, ranging from their nutritional contribution to food preservation [13]. Thanks to their production of a variety of bioactive metabolites, LAB can enhance the flavour, texture, and nutritional qualities of fermented foods [16,17,18]. They also enhance product safety by inhibiting undesirable microorganisms through the production of antimicrobial metabolites such as organic acids, hydrogen peroxide, carbon dioxide, and bacteriocins [19,20,21].

Recent investigations on Tunisian traditional fermented foods have revealed a significant diversity of LAB genera, including *Lactiplantibacillus*, *Levilactobacillus*, *Lactococcus*, *Pediococcus*, *Enterococcus*, *Leuconostoc*, and *Carnobacterium* [22,23,24,25]. The biotechnological applications of these strains extend beyond food fermentation, encompassing the development of functional foods and natural preservatives, as well as the production of bioactive compounds [22,26,27]. For example, *Streptococcus thermophilus*, widely used in dairy fermentations, not only contributes to texture and flavour development but also enhances the nutritional and functional value of fermented products by producing bioactive peptides and modulating the gut microbiota [28]. In this context, the present review provides a comprehensive and up-to-date overview of the diversity of lactic acid bacteria isolated from traditional Tunisian foods, with a focus on their functional properties and applications. It examines the main categories of Tunisian fermented foods as sources of LAB, the dominant species reported, the methods used for their isolation and identification, and their key technological and functional traits. The objective of this review is to critically assess current knowledge of LAB associated with Tunisian fermented foods and to highlight their potential roles in enhancing food quality, safety, and innovation within food systems.

## 2. Overview of Tunisian Traditional Fermented Foods

Over the last few decades, technological advancements have led to the rapid industrialisation of various food fermentation processes [29]. These advancements have enhanced the safety, shelf life, and nutritional compositions of these products [30]. As a result, many traditional products have been transformed into large-scale industrial production using commercial industrial microbes, with an important focus on quantity. In this section, Tunisian traditional fermented foods are highlighted, along with artisanal processing methods.

### 2.1. Dairy Fermented Products

Dairy products have been consumed since antiquity and remain a pivotal part of the global diet. Traditionally, these products are produced through spontaneous fermentation driven by indigenous microorganisms, particularly LAB [1]. Among these foods, *rayeb* is widely consumed in Tunisia; it is a fermented dairy beverage obtained through spontaneous fermentation of cow’s or goat’s milk, achieved by leaving raw milk at ambient temperature for at least one day, depending on the season (temperature) [31]. The product can be consumed directly or further processed into leben [32]. Indeed, leben is the aqueous fraction after fat separation of rayeb; after churning rayeb for about 40 min in a leather bag called “chekwa” (*checoua*), the mixture is separated into an aqueous fraction called leben and a fat fraction called “zebda” (butter) [33]. *Zebda* or “zebda beldia”, an artisanal butter, is a fatty dairy derivative. The churning process is crucial for making *zebda*; water can be added to improve the separation of *zebda* and *leben* [34]. Traditional butter has a limited shelf life and should be consumed within a few days [4]. In contrast, *smen* (“d’hen”) has a longer shelf life and can be stored for up to 6 months. The traditional process of smen involves washing raw butter (*zebda*) in warm water, decanting it, and then replacing the water with fresh, salted water. This process is repeated until the rinse water runs clear. The butter is then salted and stored in a clay pot ready for consumption [35].

Among traditional cheeses, two fresh cheeses are the most popular in Tunisia: “Testouri cheese” and “rigouta”. “Testouri” originates from the *Testour* region in northern Tunisia [36,37]. *Testouri* is a fresh soft cheese, traditionally made from both goat and sheep milk. It is obtained through milk coagulation using rennet, followed by whey drainage. The curd is further stored in brine to enhance flavour [38]. *Testouri* cheese has been reported to exhibit probiotic potential associated with the presence of *Enterococcus faecalis* strains [25]. *Rigouta* cheese is a traditional Tunisian fresh cheese made from cow’s milk and similar to the Italian *ricotta*. It is a soft cheese with a shelf life of 2–3 days. It is traditionally produced in the *Béja* region and involves spontaneous fermentation of fresh milk at room temperature for 1–2 days, followed by heating to coagulate the protein. The obtained curd is further drained to obtain fresh cheese [4].

### 2.2. Plant-Based Fermented Products

As part of Tunisian gastronomic cultural heritage, a wide variety of plant-based fermented products are traditionally consumed, including cereal- and vegetable-derived foods. Among vegetable-based products, fermented olives are widely consumed across the Mediterranean region [39]. Olive fermentation is an ancient and widespread practice in the Mediterranean region and is traditionally achieved through spontaneous fermentation in salted water, sometimes with the addition of aromatic plants to enhance flavour [40]. Caper berries are the fruits of the *Capparis* species, cultivated mainly in the Mediterranean basin. In Tunisia, caper fruits are fermented into pickled capers. Generally, capers are fermented in small-scale enterprises or at home using grandmother's recipes; the main procedures include washing, immersion in water for 3 days (with the water changed daily), storage in a glass jar with alternating layers of salt and capers for one week, a further washing step, and then leaving the capers to ferment for one week at room temperature [41]. *Legmi* also represents a traditional plant-based beverage in Tunisia, particularly in the southern regions. It is a fresh sap collected from the trunk of the date palm (*Phoenix dactylifera* L.), which is consumed fresh or subjected to spontaneous fermentation. Owing to its high sugar content (92–95% on a dry matter basis), *legmi* undergoes rapid fermentation by indigenous microflora, making it a potential source of lactic acid bacteria in traditional Tunisian diets. Similar fermented palm sap beverages are reported worldwide, including *toddy* in Sri Lanka, *tuba* in the Philippines, and *tuak* in Indonesia [42,43].

Cereal-based fermented products also constitute a significant part of the Tunisian diet. Common examples include couscous, traditional fermented bread, and *frik* [44]. Wheat-based foods remain predominant in Tunisia, particularly in the form of traditional breads; typical examples include *tebouna*, *mtabga*, and *meloui*. These cereal-based products are prepared from wheat flour doughs that undergo short fermentation or resting periods, allowing the development of characteristic texture and flavour through the activity of yeasts and lactic acid bacteria such as *Latilactobacillus curvatus*, *Pediococcus pentosaceus*, and *Pediococcus acidilactici* [45]. “Assidat zgougou” is a sweet cream pudding consumed in Tunisia and is prepared from *Pinus halepensis* (Aleppo pine) seeds. The preparation of *assidat zgougou* requires a spontaneous fermentation of ground Aleppo pine seeds in water overnight. After fermentation, the mixture is filtered, flour and sugars are added to the juice, and the mixture is baked until solidification [46]. The use of barley-based foods remains important in northern Africa because of their nutritional and fibre content. In Tunisia, barley is used to prepare barley soup, breads, and beer. The consumption of beer is estimated to be 0.0189 L/capita/day for the total adult population (70 Kg of body weight) [47].

### 2.3. Meat- and Fish-Based Fermented Products

Fermented meats are part of traditional Tunisian gastronomy. However, only a few traditional meat products are available in Tunisia, and many are on the verge of extinction due to changes in eating habits. The most common traditional fermented meats include *keddid*, *merguez*, *ossben*, and dried anchovies [48]. The majority of these products undergo at least one traditional method of meat preservation; to preserve meat for a longer period, salting, drying, sun-drying, and the addition of herbs with antimicrobial properties are used [6,49]. Indeed, adding salt (sodium chloride) significantly reduces water activity, and salt is considered antimicrobial because it interferes with the ability of bacterial cells to maintain internal water pressure, while the drying process accelerates moisture loss and decreases water activity [50]. *Kadid* is a dried, salted meat typical of the Maghreb (Tunisia, Algeria, and Morocco). In Tunisia, *kadid* is commonly prepared from sheep meat and fermented without starter cultures, relying on endogenous microbial flora for fermentation and preservation, although processing practices may vary between regions [6]. *Merguez* is a well-known traditional Tunisian meat product, known worldwide as a sausage [48]. It is typically made from beef or lamb, combined with sheep tail fat (approximately 20–30% of the total meat), seasoned with salt and various spices, including black pepper, hot red pepper, and paprika, and then stuffed into natural sheep casings. Similar to *kadid*, the Merguez sausages are further dried in the open air and then stored in containers filled with olive oil. Camel meat can be used for fresh and dry-fermented sausages [51,52]. Dried, salted anchovies are a traditional food in Tunisia, processed through a combination of salting and drying. Methods of salting and drying will determine the characteristics of the end product [53]. *Ossban* is a typical Tunisian dry-fermented meat prepared with sheep intestines and meat, mixed with spices and salt, and then dried in the sun for several days [54]. Overall, Tunisian foods are carriers of beneficial microorganisms and play essential roles in human nutrition and health. Scientific studies have consistently shown that LAB constitute the dominant microbial group in Tunisian fermented foods, highlighting their pivotal role in fermentation processes [20,55,56,57].

## 3. Tunisian Fermented Foods as Sources of LAB

### 3.1. Methods for Isolation and Identification of LAB from Tunisian Foods

Isolation and identification of LAB are essential approaches for understanding their diversity, technological significance, and functional roles within fermented food matrices [58]. Methods used to study LAB have changed over time, progressing from culture-dependent methods to more advanced molecular and omics-based approaches as microbiological tools and technologies have developed. This evolution has improved the accuracy and ecological relevance of LAB characterisation, particularly in complex matrices such as fermented foods [59]. In the context of Tunisian fermented foods, the majority of studies summarised in Table 1 employed de Man, Rogosa, and Sharpe (MRS) agar as the primary medium for LAB recovery. MRS is widely recognised for its capacity to support the growth of *Lactobacillus* species and other acid-tolerant lactic acid bacteria [60], and it has been applied across a variety of matrices, including raw camel milk [61,62,63,64], fermented olives [65], kadid [20,66,67], artisanal butter [68], and date palm sap (“Legmi”) [43]. To expand the spectrum of isolated LAB, some studies combined MRS with M17 or Elliker agar, particularly to favour the growth of coccoid species such as *Lactococcus* and *Enterococcus* [46,69]. Incubation conditions in Tunisian studies were carefully tailored to both the matrix and the target microbial groups. Temperatures generally ranged from 30 to 37 °C, with incubation periods spanning 24–72 h under aerobic, anaerobic, or microaerophilic conditions. Anaerobic incubation was preferentially applied for dairy-derived samples, especially camel milk, to promote the growth of lactobacilli [61,62]. Additionally, enrichment steps in liquid MRS or M17 media were occasionally performed prior to plating, particularly for fermented olives and cereal-based products, to increase the recovery of LAB from solid matrices and, in some cases, from samples with low initial bacterial counts [45,46,65,70].

Overall, classical culture-based methods remain a practical and widely used approach. However, they have some limitations. Besides being time-consuming, these methods inherently exclude viable but non-culturable (VBNC) bacteria, potentially underestimating total microbial diversity by providing only a partial representation of LAB diversity. This limitation highlights the importance of integrating complementary molecular and genomic tools to capture the full extent of LAB diversity [71,72].

**Table 1:** Diversity of LAB in tunisian traditional fermented foods: isolation, culture conditions, and identification methods.

Tunisian Foods	Principal LAB Species	Isolation and Culture Conditions	Identification Methods	Ref
Raw milk	*Lactococcus lactis* subsp. *lactis*, *Lactiplantibacillus plantarum*, *Lactiplantibacillus pentosus*, *Leuconostoc mesenteroides*, and *Levilactobacillus brevis*	MRS/M17/Elliker agar, 7 °C, 10 d	- Phenotypic identification - Molecular identification: Amplification of 16S/23S spacer regions	[69]
Camel milk	*L. plantarum* and *Limosilactobacillus fermentum*	MRS agar, 37 °C, 48 h, Anaerobic	- Phenotypic identification, - Molecular identification: 16S rRNA gene sequencing	[61]
Camel milk	*L. plantarum*	MRS agar, 30 °C, 48 h, Anaerobic	- Molecular identification: 16S rRNA gene sequencing	[62]
Camel milk	*Lactococcus lactis*, *Lactiplantibacillus pentosus*, *Lactiplantibacillus plantarum*, *Levilactobacillus brevis*, and *Pediococcus pentosaceus*	MRS agar, 30 °C, 24–48 h	Biochemical identification API systems	[63]
Camel milk	*Enterococcus faecium*	MRS agar, 37 °C, 24 to 48 h, aerobic	- Phenotypic identification, - Molecular identification: 16S rRNA gene sequencing	[64]
*Lben*	*Lacticaseibacillus paracasei*	MRS agar, 37 °C, 24–48 h	- Phenotypic identification, - Molecular identification: MALDI-TOF + 16S rRNA sequencing	[73]
*Raieb*	*L. lactis* subsp. *Lactis*, *Leu. mesenteroides*, *Streptococcus thermophilus*, *Lactobacillus delbrueckii* (subsp. *bulgaricus* and subsp. *delbrueckii*), *Lacticaseibacillus acidophilus*, and *E. faecium*	MRS agar, 37 °C, 2–3 d	- Phenotypic identification - Molecular identification: 16S rRNA gene sequencing	[74]
Testouri cheese and Rigouta	*E. faecalis*	MRS, 37 °C, 48 h, anaerobic	- Phenotypic identification, - Molecular identification: MALDI-TOF MS + species-specific PCR assay	[25]
Rigouta	*L. lactis*, *E. faecalis*	M17 agar, 30 °C, 2–3 d	- Phenotypic identification - 16S rRNA gene sequencing	[37]
Tunisian artisanal butter	*Lacticaseibacillus paracasei*	Bromo–Cresol Purple agar for isolation of LAB	- Phenotypic identification, - Molecular identification: RAPD-PCR + 16S rRNA gene sequencing	[68]
Traditional salted dried meat Kadid	*E. faecium*	MRS agar, 30 °C, 48–72 h.	Molecular identification: PCR-RFLP, 16S–23S rRNA ISR, and species-specific PCR	[66]
Kadid	*Staphylococcus xylosus*	Mannitol Salt Agar, 30° C, 48 h	- Biochemical methods (API ID 32 STAPH system), - Species-specific PCR	[56]
Kadid	*Lactiplantibacillus plantarum*	MRS agar	Biochemical and molecular identification (species-specific PCR)	[20]
Kadid	*E. faecium*	MRS agar; anaerobic incubation at 30 °C for 48–72 h	Phenotypic identification and ribosomal DNA-based methods (16S–23S rRNA intergenic spacer (ISR) PCR, RFLP analysis, species-specific PCR	[67]
Fermented olive	*L. brevis*, *L. plantarum*, and *Lactiplantibacillus pentosus*	Enrichment in MRS broth followed by plating on MRS agar and incubation at 37 °C for 48 h	Biochemical identification (API 50 CHL) and molecular identification based on 16S rRNA gene sequencing	[65]
Spontaneously fermented tomato fruit	*Leuconostoc mesenteroides*, *Lactiplantibacillus plantarum*, *Lactiplantibacillus paraplantarum*, *Enterococcus durans*, and *Enterococcus faecium*	MRS agar, 30 °C for 48–72 h	Intergenic Transcribed Spacer (ITS)-PCR fingerprinting (16S–23S rRNA); 16S rRNA gene sequencing	[75]
Fermented Seeds (“Zgougou”) from Aleppo Pine	*L. plantarum*, *L. paraplantarum*, and *E. faecalis*	Enrichment in MRS or M17 broth followed by plating on MRS or M17 agar; anaerobic incubation at 37 °C for 24–48 h	Molecular identification via RAPD-PCR and 16SrRNA gene sequencing	[46]
Cereal grains	*Latilactobacillus curvatus*, *Companilactobacillus farciminis*, *Lactiplantibacillus nantensis*, *Pediococcus pentosaceus*, and *Pediococcus acidilactici*	Enrichment in modified MRS broth (mMRS) followed by plating on mMRS agar + cycloheximide (0.1 g/L); anaerobic incubation at 30 °C for 48 h	Phenotypic identification Molecular identification by RAPD-PCR and 16S rRNA gene sequencing	[45]
Flours	*Weissella cibaria*, *Lactiplantibacillus plantarum*, *Levilactobacillus brevis*, *Pediococcus pentosaceus*, *Pediococcus acidilactici*, *Enterococcus faecium*, *Enterococcus casseliflavus*, and *Enterococcus faecalis*	Enrichment in mMRS followed by plating on mMRS agar with 0.0025% of bromocresol green and 0.01% cycloheximide; anaerobic incubation at 30 °C for 48 h	ITS-PCR	[70]
Date Palm sap “Legmi”	*L. delbrueckii* subsp. *delbruckii* and *L. mesenteroides*	MRS + cycloheximide 0.005%, 30 °C, 2 d	API galleries (API 50CH^®^ system)	[43]

Several Tunisian studies relied on classical phenotypic and biochemical identification, including morphology, Gram staining, enzymatic activities, and carbohydrate fermentation profiles using the API 50CH system. For instance, API 50 CHL galleries were used for LAB isolates from camel milk [63] and fermented olives [65], while the API 50 CH system was also utilised for isolates from date palm sap [43].

Biochemical profiling provides standardised, reproducible results and enables comparison across studies. However, its discriminatory power is limited for closely related species or newly reclassified taxa. This is particularly relevant for members of the former *Lactobacillus* genus, including *Lactiplantibacillus plantarum* and *Levilactobacillus brevis*, whose carbohydrate fermentation patterns overlap, potentially leading to misidentification. Consequently, reliance solely on phenotypic and biochemical methods may contribute to discrepancies in reported LAB diversity across Tunisian studies. To overcome the limitations of phenotypic and biochemical approaches, molecular identification has increasingly been adopted. In Tunisian fermented foods, 16S rRNA gene sequencing is the most commonly used method for LAB identification, providing reliable genus- and species-level assignments. Several studies have complemented 16S rRNA analysis with species-specific PCR assays, ribosomal DNA-based fingerprinting, or PCR-RFLP of the 16S–23S intergenic spacer region (ISR), improving discrimination between closely related taxa, such as *Enterococcus faecium* strains in *kadid* [67].

Moreover, few studies have applied proteomic approaches, notably MALDI-TOF MS, for rapid and accurate LAB identification. For instance, MALDI-TOF MS enabled precise identification of *Lacticaseibacillus paracasei* L2 from leben [73] and *Enterococcus faecalis* OB14 and OB15 from traditional cheeses [25]. While such integrated strategies enhance accuracy, most Tunisian studies remain reliant on single-gene sequencing approaches, which may not capture intra-species diversity or functional variation. This methodological constraint shapes reported LAB diversity, often highlighting robust, fast-growing species such as *Lactiplantibacillus plantarum*, *Enterococcus faecium*, *Lactococcus lactis*, and *Levilactobacillus brevis*, while underestimating low-abundance or slow-growing taxa. The application of next-generation sequencing (NGS) approaches, including 16S rRNA gene sequencing, shotgun metagenomics, or whole-genome sequencing (WGS), remains limited for Tunisian traditional fermented foods. Recently, Bedhiaf-Romdhani et al. [76] applied 16S rRNA gene NGS to profile the bacterial microbiota and molecularly characterise lactic acid bacteria (LAB) in Tunisian raw camel milk. This study not only provided a comprehensive view of dominant and low-abundance LAB populations but also enabled the first detection of *Enterococcus bulliens* in Tunisian camel milk [76], a species previously undetected through conventional culture-based analyses [61,62,63]. Such findings underscore how NGS overcomes the limitations of traditional methods that are biased toward fast-growing or easily culturable strains, often missing VBNC or low-abundance LAB.

Overall, combining culture-dependent and culture-independent approaches is essential, as it allows a more complete characterisation of the microbiota, improving our understanding of LAB diversity in Tunisian fermented foods and enhancing the potential applications of these microbial communities [77].

### 3.2. Diversity of LAB in Tunisian Fermented Foods

**Figure 1:**
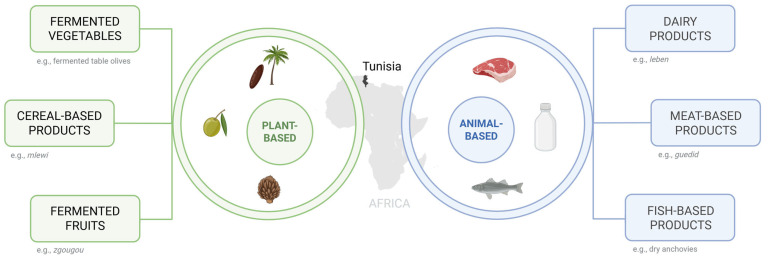
The main categories of the Tunisian traditional fermented foods. Created in BioRender. Capozzi, V. (2026) https://BioRender.com/dbyoc3e.

Traditional Tunisian fermented foods are mainly driven by the metabolic activity of LAB, which naturally occur in both plant- and animal-based matrices (Figure 1). Several studies have highlighted the dominance of LAB across a wide range of Tunisian traditional fermented products, including fermented vegetables, dairy products, meat products, and cereal-based foods. These products exhibit remarkable microbiological diversity, with species belonging to the genera *Lactobacillus*, *Lactiplantibacillus*, *Lactococcus*, *Streptococcus*, *Pediococcus*, *Leuconostoc*, *Weissella*, *Bifidobacterium*, and *Enterococcus* (Table 1) [25,45,51].

#### 3.2.1. Dairy Products

Dairy products have been studied for decades and have been exploited to isolate and screen new LAB strains. In fact, milk and milk products were considered the primary source of LAB. It is possible to trace the first milk isolation study by Liser in 1878, using rinsed milk [78]. Up to now, LAB have been isolated from raw milk, e.g., cow’s milk [79], goat’s milk [80], human milk [81], sheep milk [82], camel milk [76], and donkey milk [83], and from milk products such as cheese, butter, milk powder, yoghurt, and fermented milk [84,85]. The microbial population of dairy products depends on several factors, e.g., milk origin, environmental conditions, hygienic conditions during processing, and the manufacturing process [86]. A recent study conducted by Zammouri et al. [87] in Tunisian arid lands confirmed these variations by comparing camel, goat, and sheep milk. The authors reported that camel milk had higher counts of lactic acid bacteria (LAB) than goat and sheep milk [87].

In line with worldwide findings, Tunisian dairy products are considered a major source of LAB, with numerous publications reporting results in the scientific literature. It was confirmed that in this geographic area, the predominant microbiota in dairy products relies mostly on *Lactococcus lactis* subsp. *cremoris*, *Lactococcus lactis* subsp. *lactis*, *Lacticaseibacillus casei*, *Lacticaseibacillus paracasei*, *Limosilactobacillus fermentum*, *Lactobacillus helveticus*, *Lactiplantibacillus plantarum*, *Enterococcus faecium*, and *Enterococcus faecalis* [88].

Milk is averagely composed of 4–5% lactose [89], which can be converted into lactic acid by *Lactobacillus delbrueckii* subsp. *bulgaricus* and *Streptococcus thermophilus* via lacto-fermentation; this process acidifies the milk and prevents the growth of pathogenic spoilage/microorganisms [90]. Because of its capacity to acidify and coagulate milk, *Lactococcus lactis* MMFII, a Tunisian cheese strain, was suggested as a good starter candidate to protect fermented dairy products against listerial contamination [91].

In Tunisia, the first characterisation of a bacteriocinogenic strain was reported in 2005 by Ghrairi and colleagues for *rigouta* cheese; the results showed that lactococcin MMT24 requires the complementary action of the two peptides, pepα and pepβ, for full activity. Further, the bacteriocin produced by a *rigouta* cheese LAB strain was named lactococcin MMFII; it was the first anti-*Listeria* bacteriocin produced by a lactococcal strain [37]. Similarly, Gaaloul and colleagues identified a new bacteriocin producer, *Enterococcus faecium* GGN7 [92]. This strain produced three bacteriocins, confirmed by means of MALDI mass spectrometry analysis of the purified fractions, with molecular masses of 5471.56 Da and 4835.77 Da, corresponding to Enterocin B and Enterocin A, respectively, and a mass of 3215.5 Da. This was confirmed through microbial genome analysis. Recently, Baccouri and colleagues isolated two LAB probiotic strains from the traditional Tunisian cheeses Testouri and rigouta [25]. The two strains, *Enterococcus faecalis* OB14 and OB15, demonstrated an interesting ability to adhere to intestinal cells and reinforce the epithelial barrier [25]. Likewise, Ben Farhat and colleagues were able to characterise seven LAB strains from traditional dairy products, belonging to the species *Limosilactobacillus fermentum*, *Lacticaseibacillus paracasei*, *Lacticaseibacillus rhamnosus*, and *Enterococcus faecium*. All of the strains exhibit interesting survival rates in acidic conditions and the ability to produce antimicrobials [26].

#### 3.2.2. Plant-Based Products

Plant-based products constitute a fundamental component of the Tunisian diet, reflecting both the country’s rich gastronomic heritage and traditional food practices [6]. Among the LAB most frequently isolated from Tunisian plant-based products are *Lactiplantibacillus plantarum*, *Levilactobacillus brevis*, *Leuconostoc mesenteroides*, *Pediococcus pentosaceus*, *Pediococcus acidilactici*, *Enterococcus faecium*, and *Enterococcus faecalis* [43,45,46,65,70]. Furthermore, it was confirmed that the fermentation process method and the geographical origin could influence the microbiota [93]. For example, the lactic microbiota of fermented caper fruits showed the dominance of the *Lactiplantibacillus plantarum* group (69% of lactic microbiota), followed by *Lactobacillus brevis*, *Leuconostoc mesenteroides*, *Pediococcus ethanolidurans*, and *Enterococcus durans* (5% of lactic microbiota). Among them, four *Lactiplantibacillus plantarum* strains were identified as lactic starters due to their high salt tolerance and ability to inhibit *Escherichia coli* ATCC 10,536 and *Enterococcus faecalis* ATCC 1054 [41]. Similarly, it was reported that *Lactiplantibacillus plantarum* strains were detected in fermented olives and fermented peppers [23]. The microbial dynamics of tomato fruits during spontaneous fermentation were studied; findings revealed that spoilage bacteria and fungi disappeared after the third week of fermentation, while LAB persisted until the end of fermentation. Three LAB genera were identified, including *Lactobacillus* (old taxonomy), *Leuconostoc*, and *Enterococcus* [75]. In addition to vegetables and fruits, Tunisian cereals represent another substrate for LAB, with growing evidence supporting their relevance as sources of technologically and functionally significant strains. In a study conducted by Missaoui et al. [46] on *zgougou*, a traditional fermented product obtained from Aleppo pine seeds, the authors identified LAB strains affiliated with *Lactiplantibacillus plantarum* and *Enterococcus faecalis*, which were characterised by the absence of major virulence-related activities and by a high tolerance to acidic conditions, bile salts, and gastrointestinal enzymes. In addition to their stress resistance, these strains displayed antimicrobial, antibiofilm, antifungal, and antioxidant activities [46]. Similarly, investigations on Tunisian wheat flours have demonstrated that cereal substrates harbour LAB strains with pronounced technological traits. Mamhoud et al. [45] isolated and selected autochthonous strains such as *Latilactobacillus curvatus* MA2, *Pediococcus pentosaceus* OA2, and *Pediococcus acidilactici* O1A1, which were distinguished by their rapid acidification kinetics and notable proteolytic activity. These properties suggest a strong adaptation to cereal environments and a capacity to modulate fermentation-related biochemical transformations [45]. Complementary work by Nachi et al. [70] further highlighted the functional potential of selected LAB strains isolated from Tunisian flours. In particular, *Weissella cibaria* S25, *Pediococcus acidilactici* S16, and *Lactiplantibacillus plantarum* S28 were identified as the most promising isolates, due to their pronounced technological performance. These strains exhibited strong acidification capacity, extracellular proteolytic activity, antimicrobial effects, and notable exopolysaccharide production and antioxidant activity, underscoring the relevance of strain-level selection within cereal-associated LAB populations [70].

### 3.3. Meat and Fish Products

Meat is an excellent source of protein and contains essential nutrients for development [94]. LAB are naturally present in raw meat at relatively low levels (10^2^–10^3^ Colony-Forming Units, CFU/g) [95], which makes it an interesting source to screen LAB strains with novel/unconventional biotechnological and functional properties. Dried fish products are considered an interesting source of LAB with desirable probiotic characteristics. A recent study investigated LAB from traditionally dried and salted anchovy fish; the isolates were identified as *Enterococcus* spp. and characterised as probiotics with potential use in the meat processing industry [96].

In Tunisia, several matrices have been studied as LAB sources; we can cite sausages [7,57,97], seafood and dried meat [55], *keddid* [66], and dried *ossban* [54]. Najjari and colleagues aimed to generate a collection of *Latilactobacillus sakei* isolates representative of Tunisian meat- and fish-derived products. The food collection included seafood such as anchovies, sardines, and octopus, and meat from sheep, pork, beef, chicken, and turkey. All of the foods were processed in different ways (raw/fermented/salted/spiced/conserved in oil/smoked) and were collected from different regions of Tunisia. The amplified ribosomal DNA restriction analysis (ARDRA) and 16S rDNA sequencing identified 22 LAB isolates among 86 LAB isolates as *Latilactobacillus sakei* strains, while the remaining isolates were suggested to belong to *Lactiplantibacillus plantarum*, *Lactobacillus curvatus*, and *Pediococcus pentosaceus*, as well-documented species in meat and fish products [55]. In sausage, LAB were reported, and the incorporation of *Lactiplantibacillus plantarum* and *Pediococcus acidolactis* strains improved the quality of beef sausages, acting as bio-protective strains that extended the shelf life of Tunisian sausages [98]. Likewise, it was reported that the LAB population reached more than 10^8^ CFU/g during the ripening process of goat sausage. Inoculation with *Lactiplantibacillus plantarum* CT28 preserved the hygienic quality of the samples [96]. LAB strains previously isolated from Tunisian meat and fish products were selected as starter ferments for Tunisian dry-fermented sausage. Different combinations of starter cultures were tested, and researchers found that sausages produced with *Latilactobacillus sakei* 23K had the most desirable sensory properties [27]. Traditional fermented dry camel sausages can be an interesting matrix for analysing LAB flora; in this regard, Mejri and colleagues identified 29 isolates as *Lactiplantibacillus plantarum*. All strains showed interesting technological and safety properties, including acidification, proteolytic activity, and antimicrobial activity [99]. *Keddid* or “Gueddid” is a traditional fermented meat that can be screened for LAB strains. A total of about 50 LAB isolates were collected from the *gueddid* sample by Belgacem and colleagues, demonstrating its richness in live microbes despite the high salinity and low water activity. Among these strains, one *Enterococcus faecium* was a bacteriocin producer [92]. Dried *ossban* is a Tunisian traditional dry-fermented meat prepared from sheep intestines and meat, mixed with salt and spices, and dried for several days with exposure to the sun. The microbiological analysis of dried *ossban* showed the predominance of enterococci, mainly *Enterococcus faecium* [54].

## 4. Functional and Nutritional Properties of Tunisian Lactic Acid Bacteria with Potential Biotechnological Applications

**Figure 2:**
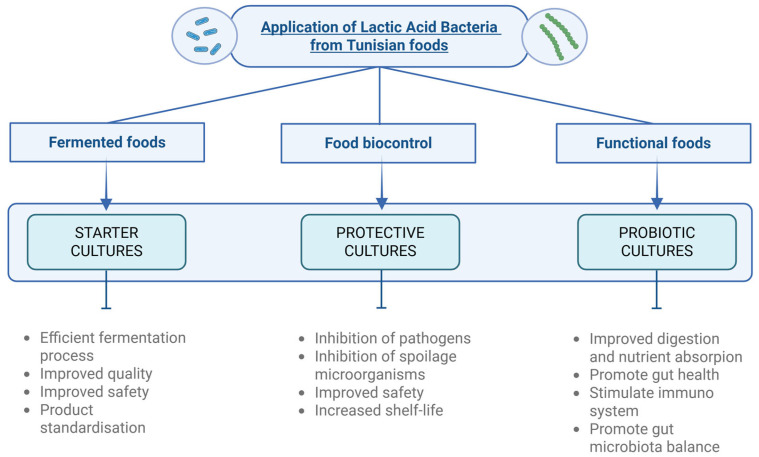
Applications of LAB strains from Tunisian traditional foods. Created in BioRender. Capozzi, V. (2026) https://BioRender.com/bo9z9pm.

LAB are widely recognised for their polyvalent role in food systems, encompassing fermentation, biopreservation, and probiotic functionality [100]. Their metabolic versatility and safety profile have positioned them as key agents in the development of traditional and innovative food products. Depending on the strain and matrix, LAB can act as starter cultures to initiate and control fermentation and as protective cultures to inhibit spoilage and pathogenic microorganisms [101] (Figure 2).

### 4.1. Food Fermentation

A vast diversity of traditional fermented foods and beverages is prepared and consumed in Tunisia. It is generally accepted that LAB are the dominant group, and their prevalence can vary depending on raw material, regional differences, and production methods. For example, it was reported that traditional vessels, including clay jars and goatskin bags (*chekoua*), could influence microbial community composition and stabilise it [102]. The effect of traditional containers: traditional vessels, such as earthenware jars and goatskin bags (*chekoua*), have been shown to influence and stabilise microbial communities. Although the specific ‘container-specific’ microbes and their interactions are not yet fully characterised, the physicochemical properties of these traditional vessels, such as porosity, micro-oxygenation, and the presence of residual microbial biofilms, likely contribute to subtle microbial dynamics that are difficult to replicate in industrial stainless-steel fermentation processes. Further studies are needed to determine whether these effects produce a unique ‘soil-like’ influence on the microbial ecosystem [103,104]. The main species involved in food fermentation include *Lactiplantibacillus plantarum*, *Lacticaseibacillus rhamnosus*, *Lacticaseibacillus casei*, *Lactococcus lactis*, *Leuconostoc mesenteroides*, and *Streptococcus thermophilus*. In milk products, strains of Lactiplantibacillus plantarum rapidly acidify through lactate production, inhibiting undesirable microorganisms. Some strains are capable of producing exopolysaccharides that enhance viscosity and consistency. While *Lacticaseibacillus rhamnosus* and *Lacticaseibacillus casei* strains contribute to the release of flavour-active peptides through proteolysis, thereby improving taste, aroma, and digestibility, *Lactococcus lactis* subsp. *lactis* and *Lactococcus lactis* subsp. *cremoris* are essential for acidification, supporting texture, viscosity, and flavour, and often act synergistically with other LAB in artisanal fermentations. *Leuconostoc mesenteroides* strains produce exopolysaccharides and aromatic compounds such as diacetyl and acetoin, enhancing consistency, mouthfeel, and the characteristic sensory profile. Finally, *Streptococcus thermophilus* ensures rapid fermentation and works in synergy with other LAB to develop taste and texture in products such as yoghurt and *rayeb*, thereby contributing to microbial stability and safety [102]. Collectively, these indigenous LAB form dynamic microbial communities that define the unique quality and organoleptic characteristics of Tunisian traditional dairy products [4]. In Tunisia, olive fermentations involve LAB, mainly *Lactiplantibacillus plantarum*, which has been isolated from local fermented olive brines. LAB drive acidification, contributing to microbial safety, while yeasts also play a role in aroma formation and de-bittering of phenolic compounds. However, specific Tunisian studies on yeasts in olive fermentation are relatively limited, while the ecological importance of *Lactiplantibacillus plantarum* in fermented vegetables has been demonstrated. Regarding fermented meats, research in Tunisia has shown that *Lactobacillus sakei* and *Staphylococcus xylosus* strains can be used as starter cultures in dry fermented sausages. LAB reduce pH and enhances safety, while staphylococci contribute to colour development and aroma establishment. A further Tunisian study confirmed the ability of the anchovy LAB strain to enhance the physicochemical and sensory properties of sausages [27]. Likewise, *Lactiplantibacillus plantarum* strains derived from traditional salted, dried meat were selected as starter cultures for fermented sausage production [20]. A recent publication by Boumaiza and colleagues reported that *Latilactobacillus sakei* could contribute to the transformation of beef into sausages with quality similar to, and even better than, that of the commercial starter [105]. Another study in this field highlighted the possible use of an *Enterococcus* strain isolated from a raw shrimp strain as a new starter, adjunct, protective, or probiotic culture in the food industry [106]. The valorisation of native microbial diversity in these traditional fermentations is essential and can pave the way for standardising production, improving safety, and maintaining the sensory characteristics typical of Tunisian heritage [107].

### 4.2. Food Preservation

LAB produce a wide range of antimicrobials, including organic acids (e.g., lactic and acetic acids), hydrogen peroxide, diacetyl, and bacteriocins, which are released as strategies to compete for nutrients and inhibit pathogens [19]. These metabolites can be exploited as bio-preservatives, either by using the producing strains as adjunct or protective cultures or by applying purified bacteriocins [108]. Such biological strategies have been shown to reduce pathogen loads in meat, dairy and vegetable matrices and to extend shelf life under mild processing conditions, offering a “clean-label” preservation alternative. In Tunisia, several studies have illustrated this potential: for instance, LAB isolated from traditional dairy products such as fermented milk and cheeses have been shown to inhibit *Listeria* and *Staphylococcus*, enhancing the microbial safety of the products [26,109]. Similarly, LAB isolated from vegetable fermentations and cereal-based sourdoughs exhibited strong antimicrobial activity, helping to limit spoilage and extend shelf life [23,45]. A study by Kraiem and colleagues evaluated the antioxidant and protective effects of intact and cell lysate preparations of *Lactobacillus pentosus* on postharvest strawberries during 10 days of storage at 4 °C. The bacterial cultures showed an interesting effect on reducing the yeast and mould on the fruit [110]. In 2022, a further study aimed to characterise the antimicrobial potential of the probiotic *Lactiplantibacillus plantarum* S61 and its application as a bio-preservative agent. Our results revealed interesting antifungal and anti-bacterial activity against yeasts (*Rhodotorula glutinis* and *Candida pelliculosa*), moulds (*Penicillium digitatum*, *Aspergillus niger*, *Fusarium oxysporum*, and *Rhizopus oryzae*), and pathogenic bacteria (*Listeria monocytogenes* ATCC 19,117, *Salmonella enterica* subsp. *enterica* ATCC 14,028, *Staphylococcus aureus* subsp. *aureus* ATCC 6538, and *Pseudomonas aeruginosa* ATCC 49,189), with inhibition zones  >10 mm [111]. In Tunisia, *Levilactobacillus brevis*, *Lactococcus lactis*, *Enterococcus faecium*, *Enterococcus faecalis*, *Enterococcus casseliflavus* and *Enterococcus mundtii* showed activity against *P. expansum*. Several *Enterococcus* strains isolated from Tunisian fermented foods, such as *E. faecalis* OB14/OB15 and GGN7, have demonstrated probiotic potential and the ability to produce bacteriocins. These functional properties make them promising candidates for use as food starter cultures. However, given that some *Enterococcus* species can be opportunistic pathogens or carry antibiotic resistance genes, strict safety assessments are necessary. The studies cited applied specific criteria, including absence of virulence genes (e.g., *esp*, *asa1*, *hyl*, and *gelE*), non-hemolytic activity, susceptibility to clinically relevant antibiotics, and, where available, a documented history of safe use in food fermentations. Such evaluations ensure that only strains meeting these safety standards are considered for application, balancing functional benefits with consumer safety [112].

A 2022 study by Rabaoui and colleagues showed that snails could be a good source of LAB strains capable of inhibiting fungal growth, forming biofilms, and tolerating gastrointestinal transit conditions. Selected *Lacticaseibacillus rhamnosus* strains from the intestinal chicken tract showed an ability to adhere strongly in vivo to intestinal epithelial cells [112]. LAB strains can be good candidates not only for bacterial antagonism but also for detoxification; a *Lactiplantibacillus plantarum* strain isolated from artisanal butter made from cow’s milk showed protective effects against cytotoxicity and genotoxicity induced by zearalenone in vivo in mice through adhesion and by decreasing its bioavailability in the gastrointestinal tract [97]. Another Tunisian study confirmed the detoxification capability of LAB strains: the *Lactobacillus kefiri* strain FR7 from dairy sources inhibited the growth of *Aspergillus flavus* and *Aspergillus carbonarius* and reduced mycotoxin production in artificially contaminated almonds and peanuts [113].

### 4.3. Food Fortification and Health Benefits

During fermentation, certain LAB can synthesise B-group vitamins; therefore, incorporating riboflavin-producing LAB into fermented foods can enhance the nutritional characteristics directly within the food matrix during fermentation and eliminate the need for exogenous fortification [114]. Additionally, some of these vitamin-producing strains exhibit probiotic properties, offering a dual advantage by simultaneously improving nutrient content and supporting gut health [16]. In Tunisia, indigenous LAB isolated from traditional dairy and cereal fermentations have been investigated for riboflavin production, demonstrating the potential to develop locally adapted functional foods with enhanced nutritional and health-promoting qualities [26,115]. Probiotic LAB can modulate the gut microbiota, strengthen gut barrier function, compete with pathogens (adhesion/co-aggregation), and interact with the immune system (stimulating anti-inflammatory responses or enhancing local immunity). Probiotic LAB can modulate gut microbiota, enhance gut barrier function, compete with pathogens through adhesion and co-aggregation, and interact with the immune system by stimulating anti-inflammatory responses or enhancing local immunity. However, these probiotic properties can vary at both the strain and species level, and LAB from different habitats may exhibit similar general functions but differ in the magnitude or specificity of their effects [30]. Strains isolated from Tunisian fermented foods have shown promising in vitro tolerance to acid and bile salts, as well as adhesion and antagonism against pathogens, key selection criteria for candidate probiotics [73]. Strains of *Lactiplantibacillus plantarum*, *Lacticaseibacillus rhamnosus*, *Lacticaseibacillus casei*, and *Lactococcus lactis* recovered from fermented dairy products such as *rayeb* and *leben* exhibit key probiotic properties, including tolerance to gastric acidity and bile salts, adhesion to intestinal epithelial cells, and antimicrobial activity against foodborne pathogens [23]. Several *Lactobacillus plantarum* strains were isolated from fermented dairy products and manifested probiotic features; for example, lactic acid bacteria isolated from Tunisian fermented dairy products exhibited a wide range of probiotic characteristics. In particular, several *Lactobacillus plantarum* LPO1, LPO2, *Lactobacillus rhamnosus* LRO1, LRO2, and *Lactococcus lactis* LLO3 strains demonstrated strong tolerance to acidic pH and bile salts, suggesting their ability to survive gastrointestinal transit. These strains also showed significant antimicrobial activity against foodborne and intestinal pathogens, mainly through the production of organic acids, hydrogen peroxide, and bacteriocin-like compounds. Moreover, strong adhesion and aggregation abilities were observed, indicating potential for intestinal colonisation and competitive exclusion of pathogens. Some isolates further displayed enzymatic activities such as β-galactosidase production, which may improve lactose digestion, as well as antioxidant properties that could contribute to host protection against oxidative stress. Together, these features highlight the probiotic potential of lactic acid bacteria derived from Tunisian fermented dairy products [73]. The probiotic *Lactiplantibacillus plantarum* strains LPO1 and LOP2, originally isolated from Tunisian camel milk, have been shown to be promising candidates for wound healing in diabetic rats [62]. Camel milk is the most similar to human milk of any other milk. Generally, it has a lower fat and saturated fatty acid content, with a higher vitamin C content, up to 10 times that of cow’s milk [116]. Another Tunisian study showed the beneficial effect of fermented camel milk containing the *Lactococcus lactis* subsp. *cremoris* strain LLO3 on the reduction in carbon tetrachloride-induced heart oxidative damage [117]. LAB strains isolated from fermented vegetables and olives have also shown similar probiotic traits, including immunomodulatory effects and competitive exclusion of pathogens [23]. The incorporation of these indigenous probiotic strains into dairy-, vegetable-, or cereal-based functional foods enables the development of locally adapted, health-promoting products that combine traditional Tunisian practices with modern nutritional benefits.

### 4.4. Key Challenges Facing the Traditional Dairy Sector in Tunisia

The traditional dairy sector in Tunisia, particularly artisanal fermented products, faces challenges including variable microbial composition, inconsistent quality, and food safety concerns. Indigenous lactic acid bacteria (LAB) from these products remain underexploited, yet they hold potential as defined starter or protective cultures [118].

Scaling up from small-scale, artisanal production to industrial manufacturing requires controlled fermentation strategies and careful integration of local ingredients to preserve the unique sensory and functional properties of traditional products. Addressing these challenges through LAB characterisation, safety assessment, and process optimisation is essential to enhance both the sustainability and industrial valorisation of Tunisia’s dairy heritage [118].

The transition from small-scale to larger-scale production and the industrialisation of grandmother’s recipes can be challenging, as it requires the intervention of several actors, including scientists, policymakers, and the food industry [119,120]. To bridge this gap, it is essential to combine cultural heritage with process engineering, biotechnological tools, and digitalisation, by transforming artisanal knowledge into data-driven, scalable workflows, and then validating these data through pilot-scale platforms [121]. In this context, protecting ancestral recipes while enabling industrial-scale production is very important and requires the intervention of policymakers and regulatory instruments (as in the case of geographical indications [GI]) [122,123]. In Tunisia, small-scale traditional fermentations, such as for olives, vegetables, and cereals, are first analysed to identify key microbial strains and communities responsible for the unique sensory characteristics. These strains can then be used as starter cultures in controlled, pilot-scale, or industrial fermentations, enabling rapid manufacturing while preserving traditional flavour, aroma, and texture.

## 5. Conclusions and Future Perspectives

Traditional Tunisian fermented foods represent a rich yet underexploited source of lactic acid bacteria (LAB) with remarkable taxonomic, functional, and technological diversity. This review has provided the first comprehensive and critical synthesis of LAB associated exclusively with Tunisian traditional fermented foods, spanning dairy, plant-based, cereal, meat, and fish matrices. Nevertheless, LAB diversity associated with wild and autochthonous plants, vegetables, and fermented products remains underexplored, despite their considerable potential as sources of strains with unique technological, biocontrol, and functional properties. Despite this recognised importance, current knowledge of Tunisian LAB remains fragmented and methodologically constrained. In fact, despite recent advances, significant knowledge gaps persist, particularly regarding the taxonomic resolution, functional diversity, safety, and technological potential of autochthonous LAB. Future investigations require a paradigm shift toward (i) the application of next-generation sequencing, whole-genome sequencing, and multi-omics strategies; (ii) comprehensive genomic safety assessments, including the evaluation of virulence factors, antibiotic resistance, and horizontal gene transfer potential; (iii) industrial scalability, through the optimisation of fermentation processes, co-culture strategies, and stability during production and storage; and (iv) the study of microbiomes as emerging resources for the sustainable transition of food systems. This is also important: the systematic integration of biological data with information on geography, environment, raw materials, and artisanal practices will enable a deeper understanding of the ecological drivers shaping LAB distribution and functionality. In general, greater preservation of lactic acid bacteria from Tunisian productions in microbial collections represents a strategic avenue in the sector.

## Data Availability

No new data were created or analyzed in this study. Data sharing is not applicable to this article.
